# A brain-penetrant bispecific antibody lowers oligomeric alpha-synuclein and activates microglia in a mouse model of alpha-synuclein pathology

**DOI:** 10.1016/j.neurot.2024.e00510

**Published:** 2024-12-14

**Authors:** Dag Sehlin, Sahar Roshanbin, Olof Zachrisson, Martin Ingelsson, Stina Syvänen

**Affiliations:** a Department of Public Health and Caring Sciences, Uppsala University, 751 85, Uppsala, Sweden; b BioArctic AB, Warfvinges väg 35, 112 51, Stockholm, Sweden; c Krembil Brain Institute, University Health Network, Toronto, Ontario, Canada; d Tanz Centre for Research in Neurodegenerative Diseases, Departments of Medicine and Laboratory Medicine & Pathobiology, University of Toronto, Toronto, Ontario, Canada

**Keywords:** Bispecific antibody, Alpha-synuclein, The blood-brain barrier, Transferrin receptor mediated transcytosis, Parkinson's disease

## Abstract

Parkinson's disease (PD) is characterized by a progressive loss of dopaminergic neurons, linked to aggregation of alpha-synuclein (αSYN) into Lewy bodies. Current treatments are symptomatic and do not halt or reverse the neurodegeneration. Immunotherapy targeting aggregated αSYN shows potential, but therapeutic efficacy is limited by poor brain penetration of antibodies. We developed a bispecific antibody, RmAb38E2-scFv8D3, based on αSYN oligomer selective RmAb38E2 fused to a transferrin receptor (TfR)-binding domain to enhance brain delivery. Both RmAb38E2 and RmAb38E2-scFv8D3 showed higher affinity for αSYN oligomers than for monomers or fibrils. *In vivo*, RmAb38E2-scFv8D3 exhibited higher brain and lower blood concentrations compared to RmAb38E2, suggesting a better brain uptake and reduced peripheral exposure for the bispecific antibody. Treatment over five days of 3–4 months old transgenic L61 mice, which overexpress human αSYN, with three doses of RmAb38E2-scFv8D3 reduced brain αSYN oligomer levels and increased microglial activation, as indicated by elevated soluble TREM2 levels. Treatment with the monospecific RmAb38E2, however, showed no significant effect compared to PBS. This study demonstrates that TfR-mediated delivery enhances the therapeutic potential of αSYN-targeted immunotherapy by resulting in a higher concentration and a more uniform distribution of antibodies in the brain. The use of bispecific antibodies offers a promising strategy to improve the efficacy of antibody therapies in PD and other α-synucleinopathies.

## Introduction

The development of effective therapies for Parkinson's disease (PD), a debilitating neurodegenerative disorder characterized by progressive loss of dopaminergic neurons, remains a significant challenge. The treatment today is based on enhancing the dopaminergic function by different strategies aimed at increasing the dopamine levels in the synaptic cleft. However, these treatment strategies are symptomatic, meaning that the underlying disease processes are not halted or reversed. The mechanism behind the degeneration of neurons in PD is not completely understood. However, the presence of pathological aggregated forms of the protein alpha-synuclein (αSYN), which eventually deposit as insoluble intracellular Lewy bodies (LB), appears to be central in the pathogenesis [[1], [2], [3]]. Immunotherapy, based on antibodies directed towards aggregated forms of αSYN, represents a promising strategy to prevent propagation of αSYN pathology. A similar approach, based on antibodies directed towards aggregated amyloid-beta (Aβ), has shown success in treating Alzheimer's disease (AD) patients by reducing Aβ brain deposits and slowing down cognitive decline [[4], [5], [6]].

A number of αSYN antibodies have been studied in clinical trials with varying results [7,8]. One reason for the inconclusive results could be that αSYN is physiologically abundant in its monomeric state, making it more advantageous to selectively target the pathological, aggregated forms of the protein. Intracellular αSYN aggregates, with Lewy bodies as the end stage, constitute the vast majority of αSYN aggregates in the brain, while a smaller fraction of soluble αSYN aggregates is believed to reside in the brain's extracellular compartment, where it would be more accessible to antibody-based therapy. Another caveat in the development of efficient immunotherapies is the low amount of antibody molecules reaching the brain. Antibodies are large molecules and do therefore display a slow and limited brain delivery across the tightly connected endothelial cells of the blood-brain barrier (BBB). However, endothelial cells of the BBB express a variety of transport proteins that ensure the delivery of essential molecules to the brain. One such transporter is the transferrin receptor (TfR), which plays an important role in the endogenous transport of iron across the BBB. Additionally, antibodies or other proteins that bind to the TfR can be transcytosed across the BBB [9,10]. During recent years, we and others have developed strategies based on TfR-mediated delivery to increase the concentration of protein drugs in the brain. For example, therapeutic antibodies can be engineered into a bispecific format to include a TfR-binding domain that facilitates brain uptake [[11], [12], [13], [14], [15], [16], [17], [18]]. The first clinical trials with bispecific antibodies directed towards Aβ and TfR are currently ongoing [19,20]. We have previously generated bispecific recombinant versions of five αSYN antibodies and shown that they display 20- to 80-fold higher brain concentrations at 2 ​h post injection compared to their monospecific versions [21]. One bispecific antibody, based on the monoclonal, oligomer selective antibody SynO2 [22] fused to two single chain fragments (scFv) of the TfR antibody 8D3 [23], RmAbSynO2-scFv8D3, was administered to aged (14–16 months) transgenic L61 mice, overexpressing human αSYN. Treatment with RmAbSynO2-scFv8D3 resulted in lower αSYN levels in the L61 mouse brain compared to treatment with the monospecific RmAbSynO2 [24].

The current study aimed to investigate the interaction between the αSYN oligomer selective antibody RmAb38E2-scFv8D3 and oligomeric αSYN in young L61 mice, at a stage where total intracellular αSYN has not yet reached levels corresponding to a full-blown pathology. These results will help us to evaluate the therapeutic potential of bispecific anti-αSYN antibody-based immunotherapy for PD and other α-synucleinopathies.

## Materials and Methods

### Antibody production and modifications

**Fig. 1:**
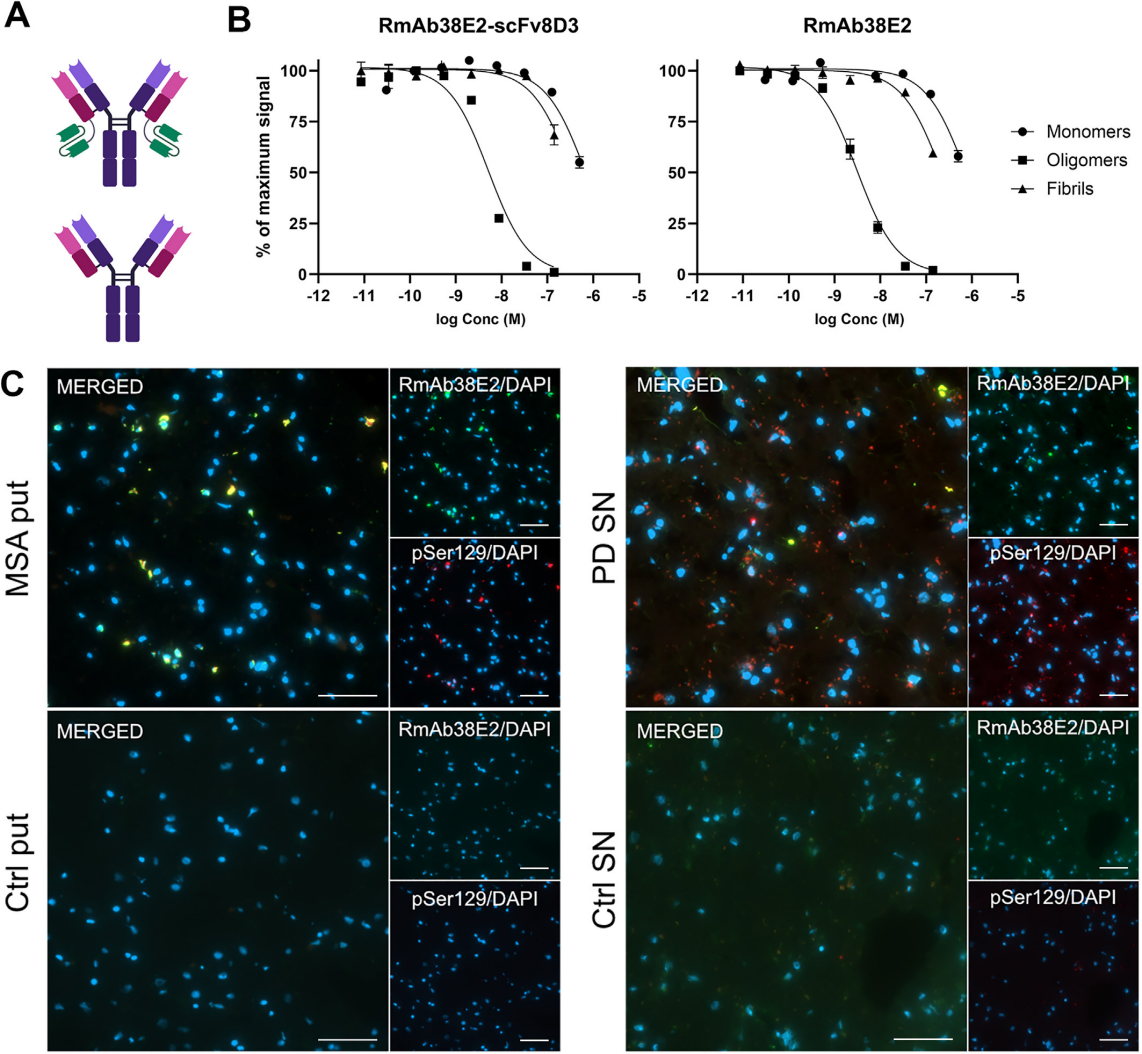
**A**. Schematic representation of the bispecific RmAb38E2-scFv8D3 (upper), with one TfR binding antibody fragment fused to the C-terminal end of each light chain, and the monospecific RmAb38E2 (lower). **B**. Inhibition ELISA analysis of RmAb38E2-scFv8D3 and RmAb38E2 binding to different αSYN species, with data normalized to the maximum signal. Both antibodies exhibited a strong preference for αSYN oligomers compared to monomers and fibrils. **C**. Co-immunostaining of RmAb38E2 (green) and pathological phosphorylated αSYN (pSer129, red) in 8 ​μm thick frozen sections from the putamen of a multiple system atrophy (MSA, left panel) patient and the substantia nigra of a Parkinson's disease (PD) patient (right panel), with a comparison to control tissue. Images were captured at 40× magnification. Scale bars ​= ​50 ​μm.

The recombinant antibodies RmAb38E2 and RmAb38E2-scFv8D3 were designed and produced as previously described [21], based on the αSYN oligomer selective antibody 38E2 [25] expressed as a mouse IgG2c. The bispecific RmAb38E2-scFv8D3 was generated by fusion of a single-chain variable fragment (scFv) of the mTfR antibody 8D3 [23] to the C-terminal end of each of the light chains of RmAb38E2 (Fig. 1). In this format, 8D3 exhibits an apparent affinity towards TfR in the low nanomolar range, which has previously been shown to be effective for brain delivery [12,26]. Both antibodies (RmAb38E2 and RmAb38E2-scFv8D3) were produced in an Expi293 cell system and purified with affinity chromatography using an ÄKTA start system (GE Healthcare AB, Uppsala, Sweden).

### Inhibition ELISA

Antibody binding to different αSYN aggregation states was assessed with inhibition ELISA, as previously described [27]. Half-area 96-well plates were coated overnight with 0.25 ng/well of recombinant αSYN (commercially available monomers, Proteos, Kalamazoo, MI, USA) or oligomers and fibrils prepared in-house as previously described [21,28], then blocked 1 ​h with 1 ​% BSA in PBS. RmAb38E2-scFv8D3 and RmAb38E2 (100 ​ng/ml) were mixed with serially diluted αSYN monomers, oligomers or fibrils and incubated in non-binding 96-well plates for 2 ​h at room temperature. The antibody-αSYN solutions were transferred to the αSYN coated plates and incubated for 15 ​min at room temperature, followed by 1 ​h incubation with horseradish peroxidase (HRP)-conjugated anti-human-IgG-F(ab’)_2_ (Jackson ImmunoResearch Laboratories, West Grove, PA, USA) and detection with K blue aqueous TMB substrate (Neogen Corp., Lexington, KY, USA).

### Immunostaining of human brain tissue

Brain sections from one patient with PD (region: substantia nigra), one patient with multiple system atrophy (MSA, region: putamen) and from one age-matched healthy control (Ctrl, regions: putamen and substantia nigra) were obtained from the Netherlands Brain Bank (NBB, Netherlands Institute for Neuroscience, Amsterdam). Written consent for tissue to be used in research had been obtained prior to death. The PD and MSA cases had been clinically and neuropathologically diagnosed, according to current guidelines. In addition, the age-matched control without signs of neurological disease or αSYN or Aβ pathology was included. Cryosections of PD, MSA and control tissue were brought to room temperature, fixed with 4 ​% PFA for 30 ​min and subjected to heat-induced antigen retrieval with 25 ​mM citrate buffer (pH 6) for 30 ​min. Sections were permeabilized in 0.4 ​% PBS/Triton-X and incubated overnight with mouse monoclonal RmAb38E2 and rabbit monoclonal anti-αSYN phospho-Ser129 antibody EP1536Y (1:250, Abcam, Cambridge, UK, ab51253). After 30 ​min incubation with fluorescently labeled anti-mouse and anti-rabbit antibodies, sections were mounted with DAPI mounting medium with DAPI (Vector Laboratories). Immunofluorescence was imaged with a Zeiss Observer Z.1 microscope and ZEN 2.6 software (Carl Zeiss Micro-imaging GmbH, Jena, Germany). Sections were washed 3 ​× ​5 min with PBS between each step.

### Animals

Three-month-old wild-type male and female mice on a B6D2F1 background (C57Bl6/DBA2) were used for measuring blood pharmacokinetics and brain uptake of the bispecific and monospecific antibodies (n ​= ​2 per antibody and time point; one male, one female). For the immunotherapy study, three- to four-month-old male and female transgenic L61 mice overexpressing wild-type, human αSYN under the Thy-1 promoter were used (n ​= ​7–8/treatment group; 2 males per group, 5–6 females per group) [29]. L61 mice were bred in-house by crossing B6D2F1 males with heterozygous L61 females on a B6D2F1 background. All mice were randomized across treatments by litters, sex and age. All experiments were conducted in compliance with the ARRIVE guidelines. All analyses, except autoradiography, were performed blinded, i.e. the person who carried out the experimental procedure had no information of whether a specific mouse belonged to the RmAb38E2-scFv8D3-, RmAb38E2 or PBS-treated group. All experiments were approved by the Uppsala Animal Ethics Committee (approval 5.8.18–13350/2017 and 5.8.18–20401/2020) and the study was conducted in accordance with the EU directive 2010/63/EU for animal experiments.

### *In vivo* experiments

Antibodies RmAb38E2-scFv8D3 and RmAb38E2 were radiolabelled with iodine-125 using the Chloramine-T method, as previously described [24,30]. Radiolabeling was always carried out within 2 ​h prior to antibody administration to the mice.

For all pharmacokinetic and *ex vivo* autoradiography studies, mice were intravenously injected with trace doses corresponding to 0.05 ​mg/kg of either [^125^I]RmAb38E2-scFv8D3 (0.75 ​± ​0.09 MBq) or [^125^I]RmAb38E2 (0.80 ​± ​0.06 MBq) formulated in PBS and sacrificed at 2 ​h, 24 ​h or 5 days post-injection. To determine the blood pharmacokinetics, 8 ​μl tail-vein blood samples were obtained at 1, 3, 6, 12, 24, 48, 72 ​h post-injection. Prior to euthanasia, a terminal blood sample was obtained from all animals, followed by transcardial perfusion with 0.9 ​% NaCl, followed by isolation of the two hemispheres, and sub-dissection of cortex, midbrain and cerebellum from the left hemisphere. Brain samples were flash-frozen, and radioactivity in the brain and blood were measured using a γ-counter (1480 Wizard™; Wallac Oy, Turku, Finland) and expressed as a percentage injected dose/g brain tissue normalized to body weight (%ID/g brain/BW and %ID/g blood/BW).

For the treatment study, all mice were intravenously injected with three doses of 10 ​mg/kg of antibody (RmAb38E2-scFv8D3 or RmAb38E2) formulated in PBS, or PBS alone, on day 1, 2 and 4, and sacrificed on day 5. In order to trace the blood pharmacokinetics of the antibodies, each dose was supplemented with trace doses of the same antibody labeled with iodine-125. Mice were injected with 0.45 ​± ​0.24 MBq of [^125^I]RmAb38E2-scFv8D3 and 0.23 ​± ​0.06 MBq of [^125^I]RmAb38E2, and 8 ​μl tail-vein blood samples were obtained 3 and 24 ​h after each injection, and a terminal blood sample was collected prior to euthanasia through transcardial perfusion with 0.9 ​% NaCl and isolation, sub-dissection and flash-freezing of brain tissue as well as radioactivity measurements as described above. The right hemisphere was kept for histological analyses. The left hemisphere was sub-dissected into cortex, the remaining part of the cerebrum (“midbrain”) and the cerebellum before the samples were kept for biochemical analyses.

### *Ex vivo* autoradiography

Sagittal 20 ​μm sections were prepared from brains isolated 24 ​h after injection of radiolabeled antibody. Sections were then exposed to a phosphor imaging plate (MS, MultiSensitive, PerkinElmer, Downers Grove, IL, USA) for seven days and scanned with Typhoon PhosphorImager (GE Healthcare). The radioactive signal was converted to a false-color scale (Royal) in ImageJ (NIH, Bethesda, MD, USA).

### Brain tissue extraction and ELISA

The sub-dissected brain regions from the left hemisphere were homogenized with a PreCellys Evolution (VWR, Stockholm, Sweden) in ice-cold Tris-buffered saline (TBS) supplemented with complete protease inhibitor (Roche, Mannheim, Germany) and PhosStop phosphatase inhibitor (Sigma, Gillingham, UK) at a 1:10 w/v ratio. The brain homogenate was centrifuged at 16 ​000 ​g for 60 ​min and the supernatant was removed and stored at −80 ​°C for further analysis. The concentration of αSYN oligomers in the TBS extracts was determined by a sandwich ELISA using MJFR-14-6-4-2 (Abcam, ab209538) as both the detection and capture antibody, as previously described [24]. The soluble microglial protein triggering receptor expressed on myeloid cells 2 (sTREM2) was also measured in TBS brain extract by ELISA using anti-TREM2 AF1729 (R&D, Abingdon, UK) as the capture antibody and BAF1729 (R&D) as the detection antibody, as previously described [31,32].

### Statistics

Statistical analyses were performed in GraphPad Prism 10.2.2 (GraphPad Software, Inc., San Diego, CA). Results are reported as mean ​± ​standard error of the mean (SEM). Statistical assessment for treatment studies was carried out by one-way analysis of variance (ANOVA) with Šídák's post hoc test for multiple comparisons; ∗p ​< ​0.05, ∗∗p ​< ​0.01, ∗∗∗p ​< ​0.001. No statistical test was used for evaluation of the pharmacokinetic profiles of the monospecific and bispecific antibodies.

## Results

Both the bispecific RmAb38E2-scFv8D3 and the monospecific RmAb38E2 (Fig. 1A) exhibited a clear preference in binding to αSYN oligomers over fibrils and monomers, with oligomer IC50 values of 5.1 ​nM and 3.1 ​nM, respectively (Fig. 1B). The IC50 values for fibril and monomer binding were 0.67 ​μM and 0.34 ​μM, respectively, for RmAb38E2-scFv8D3 and 0.72 ​μM and 0.22 ​μM, respectively, for RmAb38E2. Immunofluorescence analysis of human brain tissue sections revealed that RmAb38E2 binding co-localized with pathological αSYN in both PD and MSA brain tissue (Fig. 1C). No antibody binding was visible in control brain tissue devoid of αSYN pathology.

**Fig. 2:**
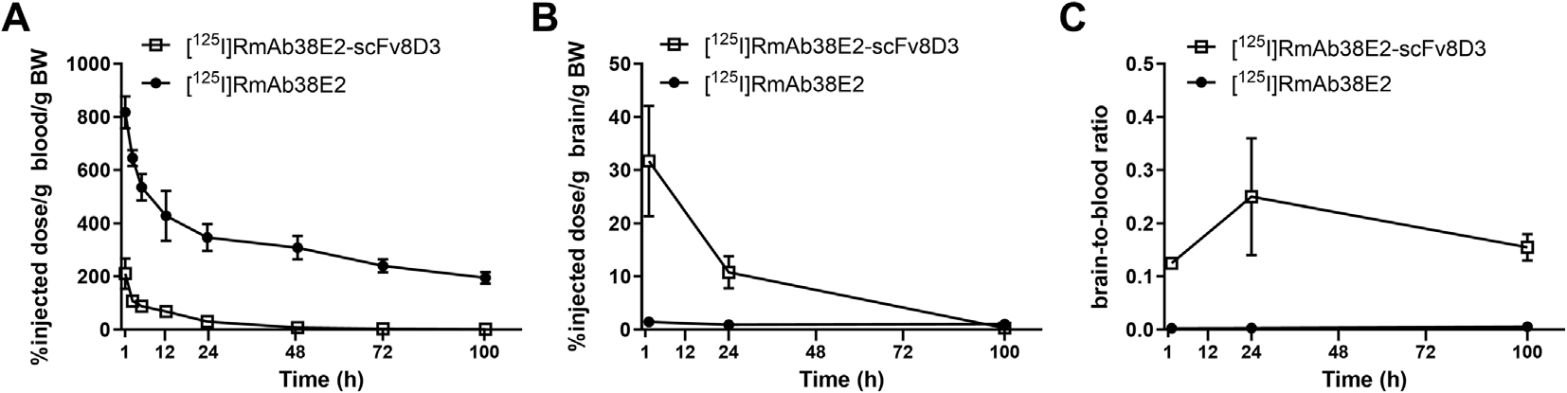
Pharmacokinetics of [^125^I]RmAb38E2-scFv8D3 and [^125^I]RmAb38E2. **A**. Blood concentrations of [^125^I]RmAb38E2-scFv8D3 and [^125^I]RmAb38E2 over time after a single trace dose (0.05 ​mg/kg). **B**. Brain levels of [^125^I]RmAb38E2-scFv8D3 and [^125^I]RmAb38E2 at 2 ​h, 24 ​h and 120 ​h after a single injection. Note, 30 ​% injected dose/g brain/g BW corresponds to 15 ​ng antibody per gram brain in mice injected with 0.05 ​mg antibody per g BW. **C**. Brain-to-blood ratio of [^125^I]RmAb38E2-scFv8D3 and [^125^I]RmAb38E2 over time. All analyses performed in wild-type mice, n ​= ​2 per antibody. All values are presented as means ​± ​SEM.

Measurement of radiolabeled antibody concentrations in blood and brain following a single injection revealed clear differences between the bispecific RmAb38E2-scFv8D3 and the monospecific RmAb38E2. At all time points, RmAb38E2-scFv8D3 exhibited lower blood concentrations compared to RmAb38E2 (Fig. 2A). However, brain concentrations of RmAb38E2-scFv8D3 were higher than those of RmAb38E2 (Fig. 2B). Consequently, due to the combination of higher brain concentrations and lower blood concentrations for RmAb38E2-scFv8D3, the brain-to-blood concentration ratio was highly elevated for the bispecific version compared to the monospecific RmAb38E2 (Fig. 2C).

**Fig. 3:**
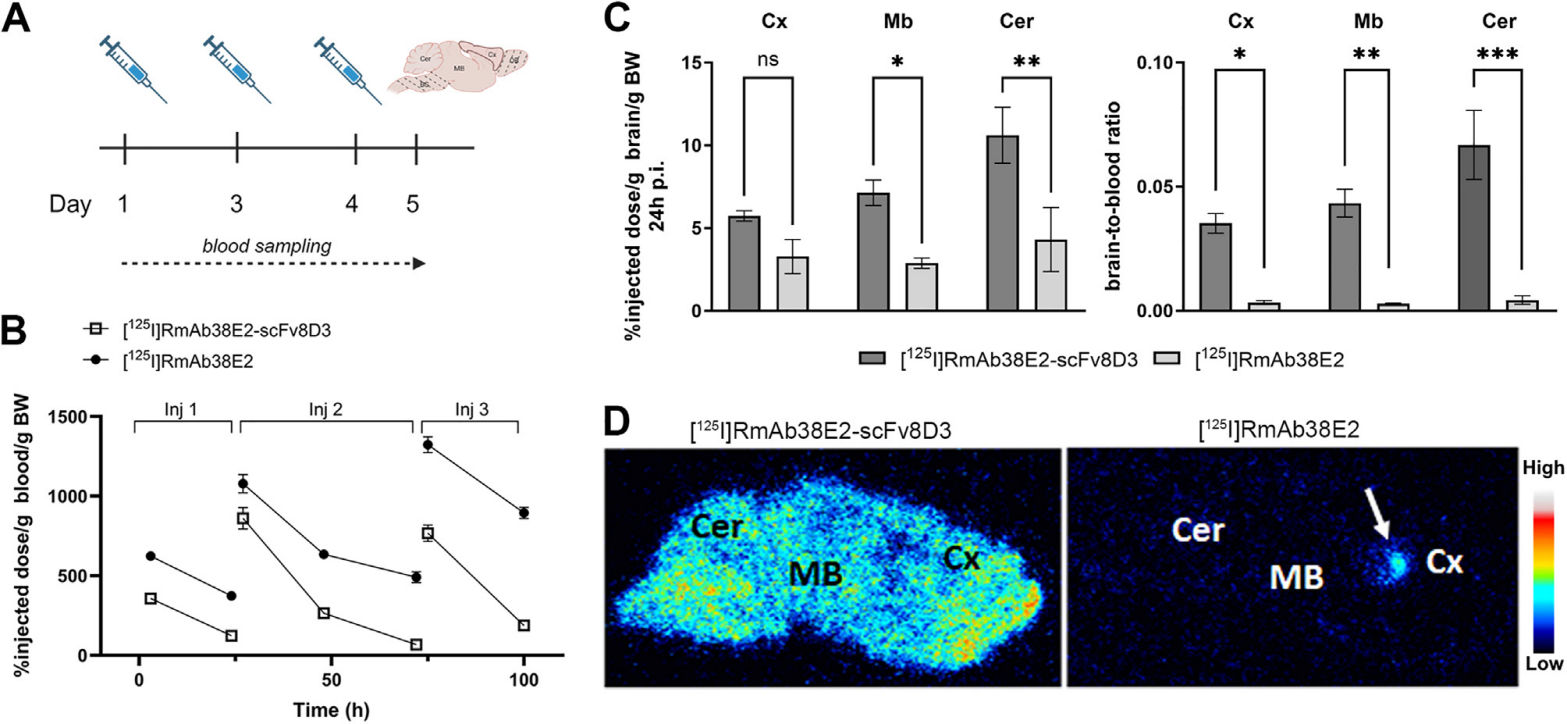
Blood and brain distribution following treatment with RmAb38E2-scFv8D3 or RmAb38E2. **A**. Study outline. 3–4 month old transgenic L61 mice were intravenously injected with either 10 ​mg/kg RmAb38E2-scFv8D3, 10 ​mg/kg RmAb38E2 (both supplemented with trace amounts of ^125^I-labeled antibody) or PBS (n ​= ​7–8/group). Mice were injected at day 1, day 2, and day 4, and were sacrificed on day 5. **B**. Blood concentrations of [^125^I]RmAb38E2-scFv8D3 and [^125^I]RmAb38E2 over time. **C**. Distribution of [^125^I]RmAb38E2-scFv8D3 and [^125^I]RmAb38E2 in cortex (Cx), midbrain (Mb) and cerebellum (Cer) (left) and brain-to-blood ratio in the same regions after study termination (right). **D**. *Ex vivo* autoradiography of 20 ​μm brain cryosections from mice treated with RmAb38E2-scFv8D3 (left) and [^125^I]RmAb38E2 (right). All values are presented as means ​± ​SEM and analyzed with one-way ANOVA followed by Šídák's multiple comparisons test. ∗p ​< ​0.05, ∗∗p ​< ​0.005, ∗∗∗p ​< ​0.001, ns ​= ​non-significant.

To assess the impact on αSYN pathology, the antibodies were administered to L61 mice via three intravenous injections on days 1, 2 and 4 (Fig. 3A). The brains were isolated on day 5. Consistent with the previous experiment in wild-type mice following a single injection, blood concentrations were higher for the monospecific RmAb38E2 compared to the bispecific RmAb38E2-scFv8D3 (Fig. 3B). Maximum blood concentration of both antibodies increased after the second and third injections. Brain concentrations of bispecific RmAb38E2-scFv8D3 were consistently higher than those of monospecific RmAb38E2 in the cortex, midbrain, and cerebellum at 24 ​h after the last antibody dose (Fig. 3C). Autoradiography confirmed higher brain concentrations of the bispecific antibody and visualized regional distribution differences between the antibodies, with a uniform distribution of the bispecific antibody while the monospecific antibody appeared to be more abundant close to the brain's ventricles (Fig. 3D).

**Fig. 4:**
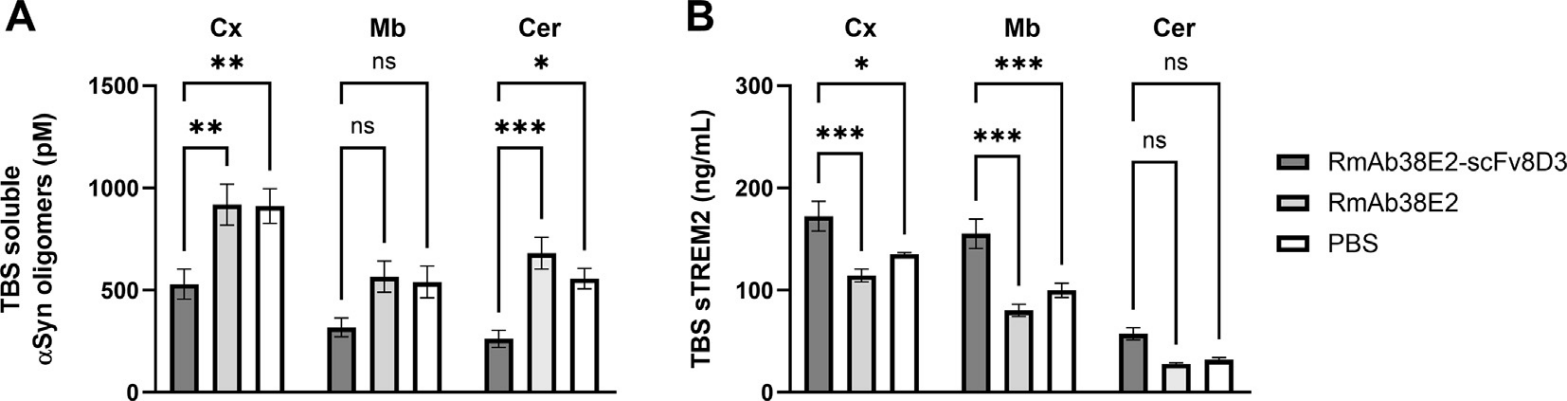
Effects of antibody treatment on levels of oligomeric αSYN (**A**) and soluble TREM2 (**B**) in TBS brain extracts prepared from cortex (Cx), midbrain (Mb) and cerebellum (Cer). N ​= ​7–8/treatment group. All values are presented as mean ​± ​SEM and analyzed with one-way ANOVA followed by Šídák's multiple comparisons test. ∗p ​< ​0.05, ∗∗p ​< ​0.005, ∗∗∗p ​< ​0.001, ns ​= ​non-significant.

The concentration of αSYN oligomers in TBS extracts prepared from the cortex and cerebellum were decreased in the L61 mice treated with the bispecific RmAb38E2-scFv8D3 compared to RmAb38E2- and PBS-treated mice (Fig. 4A). A similar trend was seen in midbrain. For the microglial marker sTREM2, the opposite pattern was observed, with increased levels in cortex and midbrain of RmAb38E2-scFv8D3-treated mice, and a similar trend in the cerebellum (Fig. 4B). In line with what was observed for αSYN oligomer levels, no difference in sTREM2 was seen between RmAb38E2- and PBS-treated mice.

## Discussion

Previous studies using models of α-synucleinopathies have demonstrated that repeated administration of antibodies against αSYN reduces brain concentrations of αSYN, and in some cases, also improves motor and cognitive functions and increases survival [[33], [34], [35], [36], [37], [38]]. In the present study, we have designed and recombinantly expressed RmAb38E2-scFv8D3, which is a bispecific recombinant version of the αSYN oligomer selective antibody 38E2, engineered to enter the brain in high concentration. In addition to the main target αSYN, RmAb38E2-scFv8D3 also binds to the TfR for facilitated brain delivery, through TfR mediated transcytosis. We first demonstrated that the addition of the extra TfR-binding antibody domain does not markedly alter either αSYN affinity or oligomer selectivity of the antibody. This is crucial for a therapeutic application, where selectivity to the aggregated, pathological forms of αSYN must be specifically targeted, while leaving the abundant physiological pool of αSYN intact.

The pharmacokinetic and brain delivery profiles were studied in a limited number of wild-type mice (n ​= ​2 per euthanasia time point) to inform the design of the subsequent treatment study in L61 mice. The small number of animals was considered sufficient, as similar antibody formats have been extensively investigated in previous studies [11,12,24,[39], [40], [41]]. The primary purpose was to confirm that the current antibodies exhibited comparable pharmacokinetic properties. Indeed, the bispecific RmAb38E2-scFv8D3 exhibited considerably elevated brain concentrations compared to the monospecific antibody RmAb38E2, consistent with findings from other bispecific antibodies of the same format [11,12,24,[39], [40], [41]]. The higher brain concentrations of RmAb38E2-scFv8D3 persisted also after repeated antibody injections, despite the difference in total blood exposure between the antibodies. While RmAb38E2 blood concentration increased after each injection, due to its slow elimination from blood, RmAb38E2-scFv8D3 returned to baseline before each subsequent injection, due to a much faster elimination from blood. Thus, the bispecific RmAb38E2-scFv8D3 showed a substantially higher brain exposure, but with a much reduced blood exposure, which is likely to reduce potential side effects related to high antibody concentrations in the periphery, which has been a problem in the development of anti-Aβ antibodies for immunotherapy of AD [19,20,42,43]. It is important to acknowledge that some of the antibody associated with the brain, contributing to the measured total brain concentration, may remain within the endothelial cells of the BBB due to its TfR binding. However, previous studies have shown that as time progresses post-administration, an increasing proportion of the antibody is found in the parenchyma relative to the endothelial cells. In fact, more than 80 ​% of antibodies with a similar bispecific format to that used in the present study are located in the parenchyma as early as 24 ​h post-injection [11]. In addition to a higher brain uptake, the bispecific antibody also displayed a much more uniform distribution across the brain, similar to what has been observed for Aβ-targeted bispecific antibodies [26,44]. Both of these features, i.e. high and uniform brain distribution, are probably necessary to sequester and degrade the scarce extracellular αSYN aggregates that reside in the brain parenchyma. Thus, the use of a brain penetrating antibody that selectively targets αSYN oligomers will ultimately produce a better therapeutic outcome than seen with traditional monospecific IgG formats devoid of facilitated brain delivery.

Due to the higher brain concentrations of RmAb38E2-scFv8D3, we hypothesized that this bispecific antibody would more efficiently reduce αSYN oligomer concentrations in the brains of young L61 mice. This pool of αSYN should contain the most soluble αSYN aggregates that can diffuse within the brain parenchyma and thus mediate toxic effects as well as spreading of αSYN pathology between cells and brain regions. Furthermore, removing soluble and diffusible αSYN pathology should also prevent the build-up of more robust pathology. Consistent with this hypothesis, we observed a decrease in oligomeric αSYN in TBS brain extracts from mice treated with RmAb38E2-scFv8D3. In contrast, treatment with the monospecific RmAb38E2 did not result in any decreased levels of αSYN oligomers compared to PBS-treated mice. A decrease in oligomeric αSYN was found in both cortex and cerebellum with a similar trend in midbrain, which again supports the notion that the widespread and uniform distribution of the bispecific antibody facilitates a therapeutic effect throughout the entire brain volume. The short treatment time in the current study will likely affect exclusively soluble αSYN oligomers. Its impact on total brain levels of αSYN or on the spreading of pathology between brain regions would occur only after a longer, chronic treatment, as observed for treatment with anti-Aβ antibodies in AD model mice [40]. However, already after three injections, we could observe an increased microglial activity, seen as an elevation of the microglial marker sTREM2. This effect was evident in both cortex and midbrain of RmAb38E2-scFv8D3 treated mice, with a trend also in the cerebellum, while RmAb38E2 showed levels comparable to PBS treated mice in all brain regions. This suggests that microglial activation is a direct and immediate response to antibody treatment and that these cells may be involved in the antibody-mediated clearance of toxic αSYN aggregates from the brain, as previously suggested. Whether this activation is sustained over time or if it changes with chronic treatment remains to be clarified in future studies.

The antibody dose of 10 ​mg/kg used in the treatment study was selected as it falls within the dose range commonly used clinically for both mono- and bispecific antibodies targeting Aβ [[4], [5], [6],^19^]. However, it should be noted that TfR saturation begins to occur at doses between 1 and 5 ​mg/kg [39,45], leading to a reduced fraction of administered antibodies reaching the brain. Despite this, the total amount of bispecific antibody delivered to the brain remains higher with increased dosing. Additionally, no sex differences were observed in any of the measured outcomes. As the study was not specifically powered to detect such differences, this aspect was not investigated further. It is important to note that the experiments conducted here cannot fully elucidate the mechanism of immunotherapy with bispecific antibodies. The observed reduction of soluble αSYN aggregates in the brain suggests the removal of a portion of the extracellular, toxic αSYN that contributes to the spreading and aggravation of the pathology. That said, studies by other researchers have demonstrated that TfR may facilitate the shuttling of bispecific antibodies across the cell membranes of neurons and astrocytes [13]. However, whether this process contributes to degradation of intraceullar deposits of αSYN remains uncertain.

In conclusion, we show that a bispecific variant of an αSYN oligomer selective monoclonal antibody, engineered to enable TfR mediated transcytosis, enters the brain in high concentrations. Further, a short-term therapeutic study with this antibody, efficiently lowers soluble αSYN oligomers in young L61 mice that express human αSYN pathology in a process that appears to be mediated by activated microglial cells. Combining the specific targeting of αSYN oligomers with TfR mediated delivery for increased brain uptake is a promising strategy to increase the efficacy of αSYN directed antibody therapies for PD and other α-synucleinopathies.

## Author Contributions

DS: Conceptualization, Data curation, Formal analysis, Visualization, Writing – original draft, Writing – review & editing. SR: Conceptualization, Data curation, Formal analysis, Visualization, Writing – original draft, Writing – review & editing. OZ: Conceptualization, Project administration, Writing – review & editing. MI: Conceptualization, Writing – review & editing. SS: Conceptualization, Data curation, Formal analysis, Project administration, Supervision, Writing – original draft, Writing – review & editing.

## Submission Declaration

The work described in this article has not been published previously or is under consideration for publication elsewhere. All authors have approved the submission of this article.

## Funding

Open access funding provided by 10.13039/501100007051Uppsala University. This work was supported by Grants from the 10.13039/501100004359Swedish Research Council (2021-01083 and 2021-03524), the Swedish Innovation Agency, 10.13039/100008444Parkinsonfonden, 10.13039/501100008599Alzheimerfonden, 10.13039/501100003792Hjärnfonden, 10.13039/501100005701Åhlén-stiftelsen, Stiftelsen för gamla tjänarinnor, 10.13039/100009673Stohnes Stiftelse, 10.13039/501100006285Magnus Bergvalls Stiftelse and 10.13039/501100006129Konung Gustaf V:s och Drottning Victorias Frimurarestiftelse. The funding bodies did not take part neither in designing the study, in collecting, analyzing, or interpreting the data, nor in writing the manuscript.

## Declaration of competing interest

The authors declare the following financial interests/personal relationships which may be considered as potential competing interests: Martin Ingelsson reports a relationship with BioArctic AB that includes: consulting or advisory. If there are other authors, they declare that they have no known competing financial interests or personal relationships that could have appeared to influence the work reported in this paper.
